# A new pseudosuchian from the Favret Formation of Nevada reveals that archosauriforms occupied coastal regions globally during the Middle Triassic

**DOI:** 10.1098/rsbl.2024.0136

**Published:** 2024-07-10

**Authors:** Nathan D. Smith, Nicole Klein, P. Martin Sander, Lars Schmitz

**Affiliations:** ^1^ Dinosaur Institute, Natural History Museum of Los Angeles County, Los Angeles 90007-4057, USA; ^2^ Institute of Geosciences, Palaeontology, University of Bonn, Bonn 53113, Germany; ^3^ Kravis Department of Integrated Sciences, Claremont McKenna College, Claremont, USA

**Keywords:** Archosauria, Pseudosuchia, Poposauroidea, Middle Triassic, Panthalassa

## Abstract

Recent studies suggest that both stem- and crown-group Archosauria encompassed high ecological diversity during their initial Triassic radiation. We describe a new pseudosuchian archosaur, *Benggwigwishingasuchus eremicarminis* gen. et sp. nov., from the Anisian (Middle Triassic) Fossil Hill Member of the Favret Formation (Nevada, USA), a pelagic setting in the eastern Panthalassan Ocean characterized by the presence of abundant ammonoids and large-bodied ichthyosaurs. Coupled with archosauriforms from the eastern and western Tethys Ocean, *Benggwigwishingasuchus* reveals that pseudosuchians were also components of Panthalassan ocean coastal settings, establishing that the group occupied these habitats globally during the Middle Triassic. However, *Benggwigwishingasuchus*, *Qianosuchus*, and *Ticinosuchus* (two other pseudosuchians known from marine sediments) are not recovered in a monophyletic group, demonstrating that a nearshore marine lifestyle occurred widely across Archosauriformes during this time. *Benggwigwishingasuchus* is recovered as part of an expanded Poposauroidea, including several taxa (e.g. *Mandasuchus*, *Mambawakalae*) from the Middle Triassic Manda Beds of Tanzania among its basally branching members. This implies a greater undiscovered diversity of poposauroids during the Early Triassic, and supports that the group, and pseudosuchians more broadly, diversified rapidly following the End-Permian mass extinction.

## 1. Introduction

Archosaurs rose to dominance in terrestrial ecosystems of the Triassic, following the End-Permian mass extinction [1]. Recent studies have inferred a rapid diversification of archosaurs and their stem lineages, which established major clades by the end of the Early Triassic [2–7]. Indeed, many lineages within Pseudosuchia (crocodile-line) archosaurs must have split by the Early Triassic due to the late Olenekian ages of the poposauroids *Xilousuchus* [8] and *Ctenosauriscus* [3]. Poposauroidea are a diverse group that was present across Pangaea and spanned the Triassic temporally. Though poposauroids are well-established as an early branching clade of paracrocodylomorphs, questions of erroneous group membership (e.g. *Diandongosuchus*, [9,10]), character-state conflict (e.g. *Nundasuchus*, [11]), and uncertain affinities of taxa from the Middle Triassic Manda beds of Tanzania (e.g. *Stagonosuchus*, [12]; *Nundasuchus*, [11]; *Mambawakalae*, [13]) have clouded patterns of relationships at the base of Poposauroidea and Paracrocodylomorpha.

Poposauroids also include a broad swath of ecomorphological diversity, including bizarre sail-backed forms, e.g. *Xilousuchus* [8,14], *Ctenosauriscus* [3], and *Arizonasaurus* [15]; edentulous bipeds, e.g. *Shuvosaurus* [16] and *Effigia* [17], and semi-aquatic members from marine settings, e.g. *Qianosuchus* [18]. A number of studies have revealed that archosauriforms exhibited a wide range of ecologies in the Middle Triassic, including numerous forms known from marine settings along the eastern [10,18,19] and western [20,21] Tethys Ocean. The occurrence of archosauriforms in marine deposits suggests these lineages evolved coastal habitat preferences soon after the Permian–Triassic extinction. Although these taxa exhibit varying degrees of aquatic adaptations, including forms (e.g. *Ticinosuchus*, *Heteropelta*) with no clear skeletal modifications departing from terrestriality, their presence in deeper marine settings has led authors to posit them as intermediate stages of marine adaptation (e.g. Steps M2–3 of [22]). Despite the Pangaean distribution of many archosauriform clades during the Middle Triassic (e.g. [4,8,10,19]), no taxa have been described from marine settings along the Panthalassan coast, a region bordering the western half of Pangaea, and rich in Middle Triassic marine fossils [23–26].

Here we describe a new pseudosuchian from the Anisian (Middle Triassic) Fossil Hill Member of the Favret Formation (Nevada, USA), which represents a pelagic setting in the eastern Panthalassan Ocean. Coupled with the broader archosauriform record from the eastern (*Diandongosuchus*, *Litorosuchus*, *Qianosuchus*) and western (*Heteropelta*, *Ticinosuchus*) Tethys Ocean, this taxon reveals that pseudosuchians were also components of Panthalassan ocean coastal settings, demonstrating that the group occupied coastal habitats globally during the Middle Triassic.

## 2. Systematic palaeontology

Class Reptilia Linnaeus, 1758

Archosauriformes [27] (*sensu* [2])

Archosauria [28] (*sensu* [29])

Pseudosuchia [30] (*sensu* [2])

Poposauroidea [31] (*sensu* [2])

Genus *Benggwigwishingasuchus*

Specific eptithet *eremicarminis*

*Benggwigwishingasuchus* *eremicarminis* **gen. et sp. nov.**

(figure 1)

**Figure 1.**
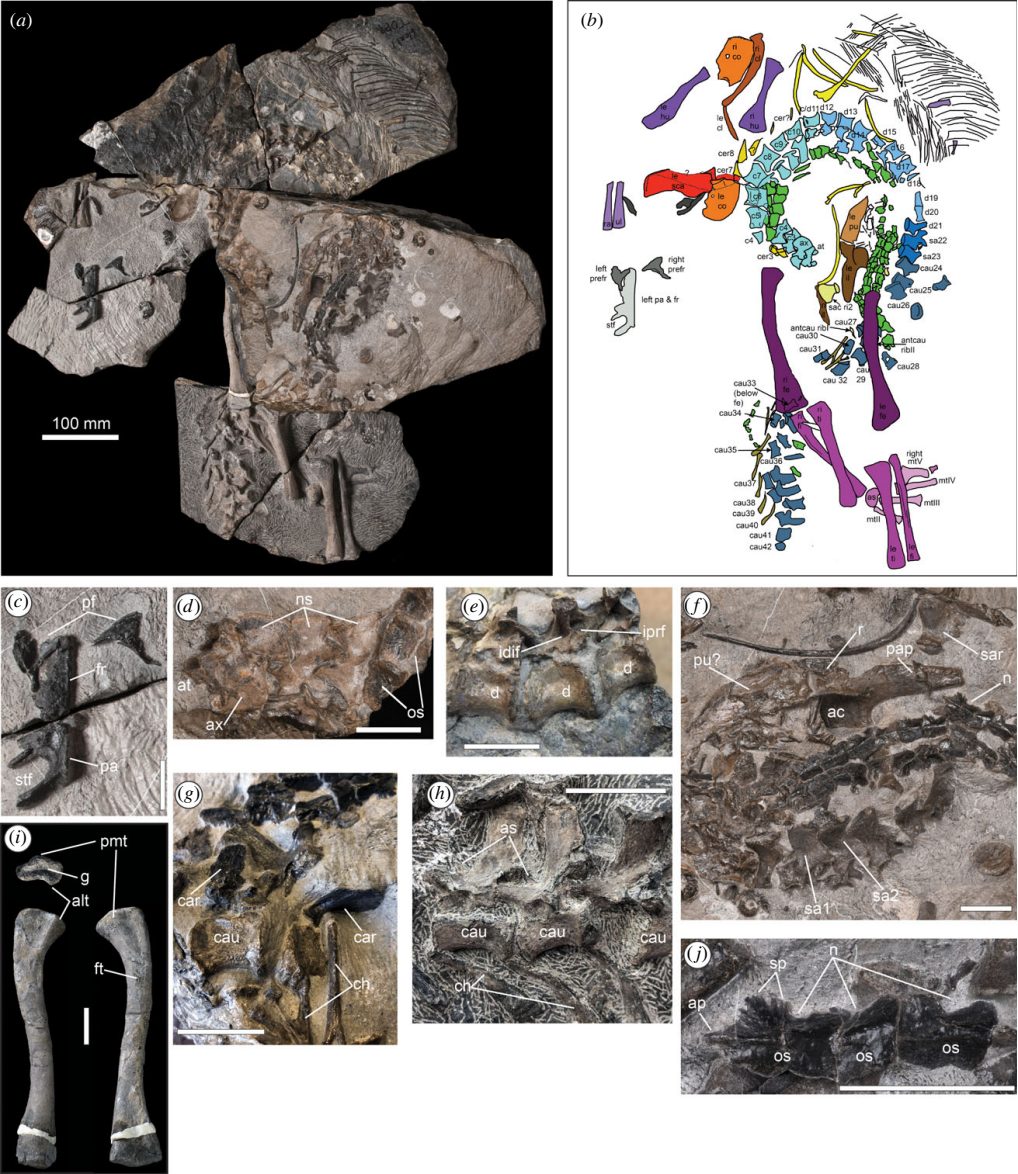
Holotype skeleton (LACM-DI 158616) (*a*) (pelvis block mirrored for articulation); and interpretive drawing (*b*) of *Benggwigwishingasuchus eremicarminis*, with insets of: skull roof (*c*), atlas/axis region (*d*), dorsal vertebrae (*e*), sacral region (*f*), proximal caudal vertebrae (*g*), mid-caudal vertebrae (*h*), right femur (*i*), and sacral region osteoderms (*j*). Scale bars, 25 mm unless indicated. ac = Acetabulum; alt = anterolateral tuber; ap = anterior process; as = accessory spine; at = atlas; ax = axis; c = cervical; car = caudal rib; cau = caudal; ch = chevron; d = dorsal; idif = infradiapophyseal fossa; iprf = infraprezygapophyseal fossa; fr = frontal; ft = fourth trochanter; g = groove; n = notch; os = osteoderm; pa = parietal; pap = postacetabular process; pf = prefrontal; pmt = posteromedial tuber; pu = pubis; r = ridge; sa = sacral; sar = sacral rib; sp = spikes; stf = supratemporal fenestra.

*Nomenclatural acts*. This publication and its nomenclatural acts are registered at ZooBank. The publication is registered under LSID urn:lsid:zoobank.org:pub:20E676DF-19CD-4DB4-863F-FFE6AC426E9C, the new genus *Benggwigwishingasuchus* under LSID urn:lsid:zoobank.org:act:1B47E656-F30B-435B-B7F6-05BFD7CEF5E6, and the specific name *B. eremicarminis* under LSID urn:lsid:zoobank.org:act:295B10E0-2026-4ECA-8878-03D212298C5D.

### (a) Etymology

The generic name combines ‘Benggwi Gwishinga’ from the Shoshone term for ‘catching fish’, with ‘suchus’, the Greek term for Sobek, the Egyptian crocodile-headed god. The specific epithet combines the Latin ‘erema’ and ‘carminis’, meaning ‘desert song’, and honours Elaine Kramer and Monica Shaffer, and their love of the palaeontology, museums, and opera of the southwestern USA. The binomen is intended to translate roughly as ‘Fisherman Croc's desert song’.

### (b) Holotype

LACM-DI 158616, a partially articulated skeleton, including some cranial elements but most of the axial column, girdles and limbs.

### (c) Locality and age

Fossil Hill Member of the Favret Formation, Favret Canyon, Augusta Mountains, Pershing County, Nevada, USA. The type locality, LACM LOC 8057, is near the top of the north slope of Favret Canyon at an altitude of 1911 m. The horizon pertains to the *Frechites occidentalis* Zone, which is late Anisian (Middle Triassic) in age [32].

### (d) Diagnosis

Mid-sized pseudosuchian (total length: approximately 1.5 m; femur length: 17.75 cm) diagnosed by the following unique combination of characters (*indicates autapomorphy, ^†^indicates autapomorphy inferred from phylogenetic analysis): semilunate eminence on middle of ventral ramus of prefrontal*; ventral ramus of prefrontal broadly exposed in lateral view, extensive ventrally and excludes lacrimal from orbit; supratemporal fossa well-exposed dorsally on parietal; high cervical vertebrae count (10–11); low dorsal vertebrae count (10–11)*; dorsal vertebrae with well-developed laminae and deep infradiapophyseal fossae; anterior caudal vertebrae with well-developed, spatulate ribs; anterior caudal ribs asymmetrical in dorsal view, with diagonal bevel on posterolateral edge*; weakly sinusoidal fibular shaft; metatarsal IV longer than metatarsal III^†^; metatarsal IV subequal in length to metatarsal II^†^; robust lateral plantar tubercle on ventral face of metatarsal V; dorsal osteoderms in one-to-one, mirrored alignment in dorsal aspect^†^; osteoderms with indentations creating an ‘hourglass’ waisting in dorsal view*; osteoderms with multiple short spikes on lateral and medial edges.

### (e) General description

The left frontal and parietal of *Benggwigwishingasuchus* are exposed in dorsal view and are unornamented, with the supratemporal fossa exposed dorsally (figure 1*c*). The left prefrontal is visible medially, and the right element laterally. The prefrontal is T-shaped with a broad contribution to the skull roof, and a long ventral ramus, likely excluding the lacrimal from the orbit, as in *Litorosuchus* [19]. A semilunate eminence projects anteriorly from the middle of the ventral ramus.

The vertebral column exhibits extreme dorsal bending, with the atlas/axis situated adjacent to the pelvis. This opisthotonic posture [33], and levels of articulation throughout the skeleton (including gastralia and osteoderms), suggests the specimen was not transported extensively post-mortem. The axis has a weakly dorsally convex neural spine in lateral aspect (figure 1*d*). Mid-cervical centra are slightly elongate relative to the dorsal vertebrae, as in Poposauroidea and Avemetatarsalia. Vertebrae lack pneumatic foramina, but the dorsal vertebrae exhibit lateral centrum depressions and well-developed neural arch laminae, including deep infrazygapophseal and infraprezygapophyseal fossae (figure 1*e*), which are common in poposauroids and avemetatarsalians. The dorsal column is unusually short, consisting of only 10–11 vertebrae. Sacral vertebrae are unfused, but it is difficult to identify positions definitively, as their centra are not visible, and only the right primordial sacral 2 rib is visible, in articulation with the ilium postacetabluar process (figure 1*f*). We identify primordial sacral vertebra 1 based on its large, sloping rib facet, confined to the anterior half of the neural arch. The posteriorly following sacral vertebra likely represents primordial sacral vertebra 2, based on the relative size of its left rib facet, in comparison to the articulation surface on the contralateral sacral rib 2. The proceeding element is considered the first caudal, but given its morphology and enlarged rib facet, it is also possible that it represents primordial sacral vertebra 2, with an ‘insertion’ representing a third sacral vertebrae, as in *Qianosuchus* [2]. The proximal caudal vertebrae are unique among archosaurs in possessing well-developed, spatulate ribs that are asymmetrical in dorsal view, with a diagonal bevel on their posterolateral edges (figure 1*g*). Several mid-caudal vertebrae have subtle accessory laminar processes on the anterior neural spines (figure 1*h*), as in Paracrocodylomorpha, and *Ticinosuchus* [2,12]. Caudal chevrons are bifurcated at their dorsal ends (figure 1*g*), as in *Qianosuchus* [18], *Poposaurus* [34] and *Gracilisuchus* [35].

Portions of the pectoral girdles are preserved primarily in part-counterpart pieces, obscuring their morphology. Both coracoids exhibit a weak postglenoid process and notch, an archosaurian synapomorphy [2]. The humerus is well expanded proximally and distally, and its length ratio to the femur is 0.59, which is short for most archosauriforms. The distal portions of the left radius/ulna are preserved in near articulation, and the end of ulna has a flattened distal surface, as in most pseudosuchians [2].

The left ilium is exposed in lateral view, with the anterior portion of the preacetabular process obscured by a portion of plate-like bone that could represent the pubis (figure 1*f*). The ilium has a crest dorsal to the supracetabular crest, as in many paracrocodylomorphs, though it has been abraded. Both hindlimbs are semi-articulated, with the right limb including metatarsals II–V. The right femur has been prepared out allowing interpretation (figure 1*i*). A small, rounded anteromedial tuber is present, as in most archosaurs [2], and a large posteromedial tuber is also present. The proximal femur has a deep, well-defined transverse groove as in many paracrocodylomorphs, and the femoral head is oriented approximately 45° to the distal condyles. The fourth trochanter is symmetrical, low and mound-shaped. The distal ends of both fibulae are symmetrical and weakly rounded, as in poposauroids [2]. The right metatarsus is well-developed, but not compacted, and also much shorter than the tibia/fibula. Metatarsal IV is slightly longer than metatarsal III, uncommon among Archosauriformes, and also longer than metatarsal II. Metatarsal V has a hooked proximal end, a robust lateral plantar tubercle ventrally (also present in *Nundasuchus*; [11]), and a well-developed phalanx V-1 which is roughly as wide as long.

*Benggwigwishingasuchus* has well-developed osteoderms throughout the vertebral column. Osteoderms extend as two paramedian rows above the vertebrae, with rows in one-to-one mirror image alignment dorsally. There are more than one set of osteoderms per vertebral element, as in Gracilisuchidae, *Ticinosuchus*, and *Qianosuchus*. Presacral osteoderms are only slightly longer than wide, with anterior processes and a lateral bend, as in many pseudosuchians [2]. Osteoderms are not present ventrally, nor along the limbs. Osteoderm morphology varies across the column, and elements above the posterior dorsals and sacrals display autapomorphies (figure 1*j*). These osteoderms exhibit numerous short spikes or crenulations along the anterior half of their medial and lateral edges, which is similar to the ‘morphotype 1’ osteoderms described in *Litorosuchus* [19], and some phytosaurs [36, fig. 21], but otherwise unique. Distinct lateral and medial indentations create an ‘hourglass’ waisting to the osteoderms in dorsal view. This osteoderm morphology has not been documented in any other archosauromorph taxon.

## 3. Methods

### (a) Institutional abbreviations

**LACM-DI**, Dinosaur Institute, Natural History Museum of Los Angeles County, Los Angeles, California, USA

### (b) Phylogenetic analyses

To assess the phylogenetic relationships of *Benggwigwishingasuchus*, we used the dataset of Butler *et al*. [13], which extensively samples pseudosuchian archosaurs, while including an array of avemetatarsalians and non-archosaurian archosauromorphs. In addition to coding *Benggwigwishingasuchus* into the dataset, we added *Diandongosuchus fuyuanensis* [9], and *Litorosuchus somnii* [19], also known from Middle Triassic marine sediments of China. We also devised a series of constraint topologies to assess alternative hypotheses of relationships (details in the electronic supplementary material).

### (c) Habitat inference

To quantitatively assess the ecology of *Benggwigwishingasuchus*, we follow an approach guided by bone compactness [37] and phylogenetic flexible discriminant analysis (pFDA; [38]; https://github.com/lschmitz/phylo.fda). We estimated bone compactness from a microCT scan of the femoral midshaft (BoneProfileR; [39]) adding the observation to Fabbri *et al*.'s [37] dataset. *Benggwigwishingasuchus* is the sister taxon to *Poposaurus* in the phylogeny of the taxa included in this analysis, and we adjusted the minimum age of the node defining Pseudosuchia + Avemetatarsalia to 247.2 million years, the beginning of the Anisian. We iterated pFDA over 100 supertrees with different branch lengths to explore the effects of variable time-calibrations, using Fabbri *et al*.'s [37] R scripts.

## 4. Results

### (a) Phylogenetic relationships

**Figure 2.**
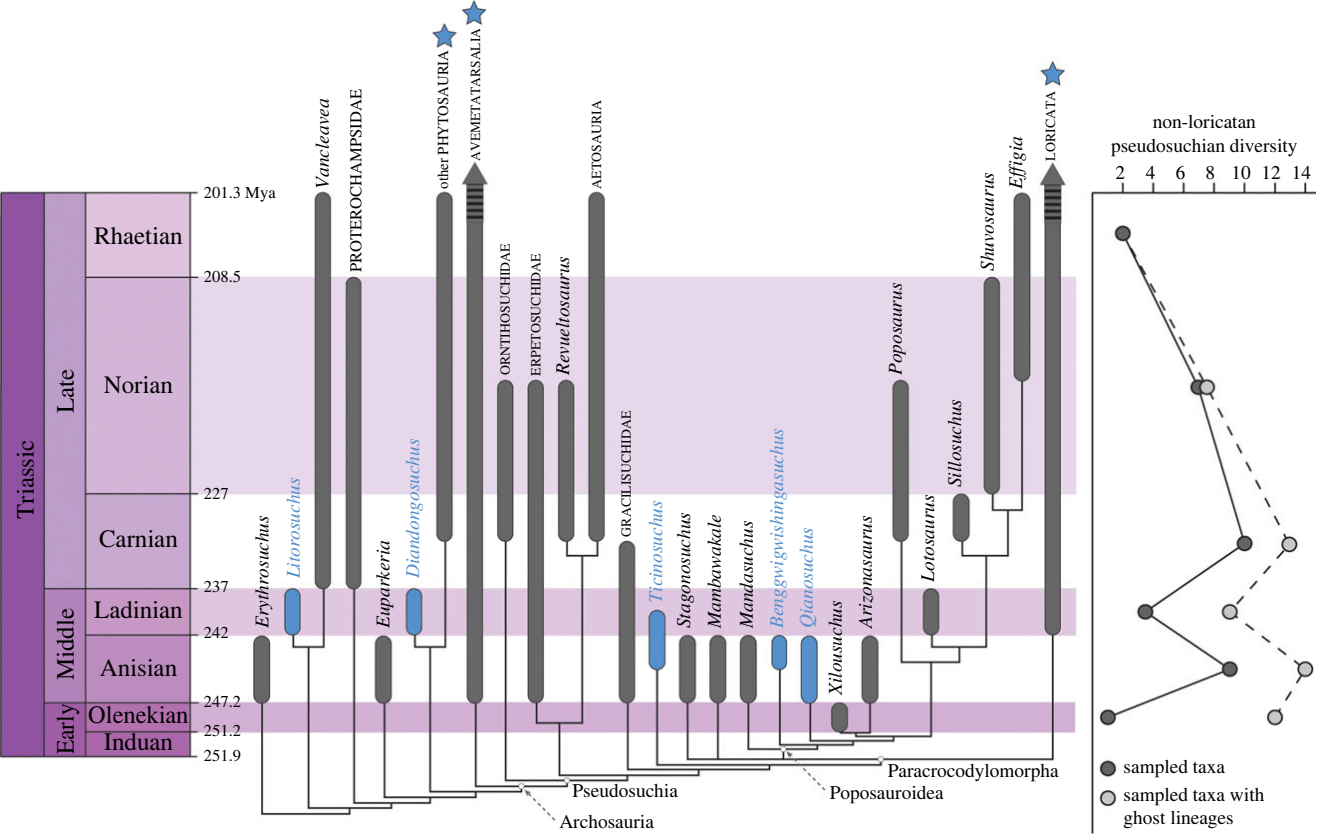
Phylogenetic relationships of *Benggwigwishingasuchus eremicarminis*. Relationships crownward of *Erythrosuchus* are shown, and several clades are condensed (see electronic supplementary material). Taxa known from marine depositional environments are indicated in blue. Blue stars indicate groups with derived taxa from marine settings. Right panel measures non-loricatan pseudosuchian diversity with and without ghost lineages counted. Timescale from Walker & Geissman [41].

The phylogenetic analysis recovered 72 MPTs of 1614 steps (CI: 0.320; RI: 0.741). A strict consensus tree is presented in the electronic supplemementary material [40], and a condensed tree is in figure 2. *Benggwigwishingasuchus* is recovered as part of an expanded Poposauroidea, which includes *Mandasuchus* as its earliest-branching member. *Benggwigwishingasuchus* is the next earliest-branching taxon, representing the sister-taxon to remaining poposauroids, which are recovered in an identical topology as found by Butler *et al*. [13]. Poposauroidea is part of a larger Paracrocodylomorpha, in a polytomy with Loricata, *Stagonosuchus* and *Mambawakale*. An Adams consensus tree of the MPTs (see electronic supplementary material [40]) reveals *Stagonosuchus* as the unstable member of this polytomy, resolving *Mambawakale* as the earliest-branching member of Poposauroidea. However, there are MPTs that recover *Stagonosuchus* within a larger Poposauroidea as well. As in Butler *et al*. [13], *Ticinosuchus* is recovered as the sister-taxon to Paracrocodylomorpha, with Gracilisuchidae as the successive sister-taxon to this larger clade. The remaining relationships of Pseudosuchia, as well as the more crownward topology within Loricata, are identical to those recovered in Butler *et al*. [13].

Characters supporting the higher-level relationships of *Benggwigwishingasuchus* include: a small, rounded anteromedial tuber on the femur (Archosauria), a nearly flat distal ulna (Pseudosuchia), an anterior process on the presacral osteoderms (Gracilisuchidae + Paracrocodylomorpha), an accessory laminar process on the middle caudal neural spines (*Ticinosuchus* + Paracrocodylomorpha), and a transverse groove across the proximal femur (Paracrocodylomorpha). Characters supporting the placement of *Benggwigwishingasuchus* within Poposauroidea include: lack of sculpturing on the frontals, relatively long mid-cervical centra, chevrons with two separate articular heads, an iliac crest dorsal to the supracetabulum, a low, crest-shaped M. iliofibularis tubercle, a symmetrical and weakly rounded distal end of the fibula, and presacral osteoderms with a bent lateral edge (see electronic supplementary material, for detailed character optimizations). The sister-taxon relationship between *Benggwigwishingasuchus* and other poposauroids is not strongly supported, with less than 50% bootstrap support, and a Bremer support value of 1. The traditional Poposauroidea clade of *Qianosuchus* plus other poposauroids recovered by Butler *et al*. [13] is supported by a 70% bootstrap value, and a Bremer support value of 2. However, deeper relationships within Paracrocodylomorpha are weakly supported, as in recent analyses [11,13,42,43].

Interestingly, *Benggwigwishingasuchus* is not recovered as a sister-taxon to, or in a monophyletic clade with, any other Middle Triassic archosauriforms known from marine sediments (figure 2). Constraint analyses suggest a monophyletic grouping of all five taxa would be 39 parsimony steps sub-optimal to the MPTs (see electronic supplementary material [40]). Enforcing a monophyletic *Benggwigwishingasuchus*, *Qianosuchus* and *Ticinosuchus* clade necessitates trees 10 steps longer than the MPTs, whereas a sister-taxon relationship between *Benggwigwishingasuchus* and *Qianosuchus* adds two additional steps, but otherwise recovers the same poposauroid relationships as the original analysis. Enforcing topologies with *Benggwigwishingasuchus* outside of Pseudosuchia or Archosauria necessitates trees seven or nine steps longer, respectively.

### (b) Habitat inference

The femoral midshaft of *Benggwigwishingasuchus* has a diameter of 16 mm and an estimated bone compactness of 0.626 (see electronic supplementary material [40]). Posterior probabilities obtained from 100 pFDA iterations suggest that *Benggwigwishingasuchus* was a non-diving animal. The median probability of being a subaqueous forager was 0.244 when performing pFDA on bone compactness by itself, and 0.243 when analysing bone compactness and the log_10_ of midshaft diameter jointly.

## 5. Discussion and conclusion

### (a) Poposauroid diversification

*Benggwigwishingasuchus* is phylogenetically nested around other penecontemporaneous Middle Triassic pseudosuchians. However, given the Early Triassic age of *Xilousuchus sapingensis* [8,14] and *Ctenosauriscus* [3], the recovery of *Benggwigwishingasuchus*, *Mandasuchus*, and possibly also *Mambawakale* and *Stagonosuchus* (all Anisian) at the base of Poposauroidea as a paraphyletic grade adds multiple ghost lineages implying a broader Early Triassic diversity of the group. Two to four additional nodal divergences, and potentially six to seven ghost lineages, occurred within basal Poposauroidea during the Early Triassic, with the lineages leading to *Benggwigwishingasuchus*, *Mandasuchus*, and possibly *Mambawakale* and/or *Stagonosuchus* extending into the Middle Triassic. Indeed, when considering ghost lineages, diversity of non-loricatan pseudosuchians peaks earlier in the Anisian, and is substantially higher in the Olenekian compared to the sampled diversity present in this stage (figure 2). This hints at a greater undiscovered diversity of Early Triassic poposauroids, and adds support that this clade, and pseudosuchians more broadly, diversified rapidly following the End-Permian mass extinction [3,4,8].

### (b) Middle Triassic pseudosuchian palaeobiogeography and palaeoecology

**Figure 3.**
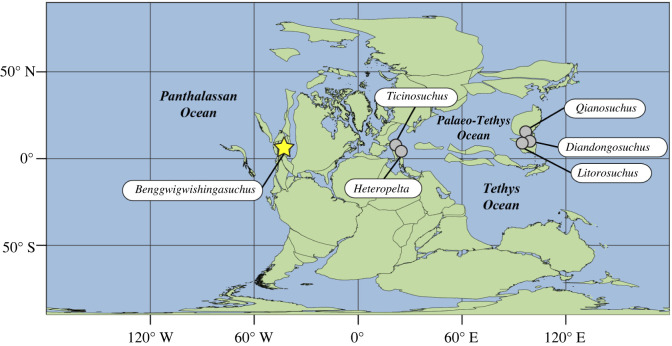
Middle Triassic palaeogeographic map with localities of Archosauriformes known from marine settings (details in the electronic supplementary material).

The Fossil Hill Member of the Favret Formation has almost exclusively yielded large-bodied ichthyosaurs, which are the dominant part of a pelagic, cephalopod-based food web [23,26]. Sediments of the Fossil Hill Member are interpreted as below wave-base shelf carbonate mudstones, deposited tens of kilometres away from the palaeo-coastline [44,45]. No shallow-water or coastal marine reptiles are known from the Fossil Hill Member with the apparent exception of *Benggwigwishingasuchus*. The discovery of *Benggwigwishingasuchus* indicates that pseudosuchian archosaurs were components of coastal settings along the Panthalassic Ocean during the Middle Triassic. The enigmatic archosaur *Sikannisuchus* from the Pardonet Formation of British Columbia [46] may suggest this Panthalassan coastal presence continued into the Late Triassic (Norian), but the relative paucity of Late Triassic marine reptile lagerstätten may limit our ability to infer how temporally and geographically widespread these patterns were. Coupled with *Qianosuchus* from the Anisian Guanling Formation of China [18], and *Ticinosuchus* from the Anisian–Ladinian [47] of Switzerland, *Benggwigwishingasuchus* reveals that pseudosuchians were occupying coastal marine habitats on a global scale during the Middle Triassic (figure 3). Broad occupation of coastal habitats by pseudosuchians is also supported by the Middle Triassic ichnological record, which includes diverse archosaur trackways from tidal flat deposits [48,49], and evidence of pseudosuchian poposauroid trackmackers [50].

As with *Qianosuchus* and *Ticinosuchus*, *Benggwigwishingasuchus* lacks many incontrovertible skeletal adaptations associated with a secondarily aquatic or marine lifestyle, though their largely articulated skeletons argue against extensive transport prior to deposition. The opisthotonic posture [33] of *Benggwigwishingasuchus* and *Qianosuchus* specimens also suggests minimal post-mortem transport. Following Motani & Vermeij [22], we posit that *Benggwigwishingasuchus* represents an M2–3 step of marine adaptation (direct feeding in the saline sea and water balance without terrestrial fresh water), similar to *Ticinosuchus* and *Qianosuchus*. This interpretation is congruent with the results of pFDA. Current histological data [51] and the humerus/femur length ratio [52] of *Benggwigwishingasuchus* are also consistent with a primarily terrestrial mode of life. Thus, we cannot exclude that *Benggwigwishingasuchus* was only an incipient user of marine resources (step M1 of [22]) and swept into the marine shelf depositional setting [33].

The fact that *Qianosuchus*, *Ticinosuchus* and *Benggwigwishingasuchus* are not recovered in a monophyletic clade implies that a coastal marine lifestyle was not a single evolutionary habitat transition characterizing one subgroup of pseudosuchians, but rather a strategy employed broadly across the group. When considering Archosauriformes, the basal phytosaur *Diandongosuchus* [10], *Litorosuchus* [19], and possibly *Heteropelta* [21], suggest that as many as six lineages were independently exploiting this niche during the Middle Triassic (figures 2 and 3). These repeated instances of ecological change may suggest that aspects of the early archosauriform body plan, physiology, or palaeoecology predisposed the group to aquatic transitions [10,53]. However, the fact that these taxa do not represent incipient members of larger secondarily aquatic marine clades also reinforces the findings of Motani & Vermeij [22], that reaching advanced stages of marine adaptation (e.g. steps M4, M5) is rare, whereas transitions to intermediate stages (e.g. steps M1–M3) is more common across tetrapod evolution. It appears that although experimentation with coastal life was ubiquitous in Triassic archosauriforms, it did not lead to broader aquatic radiations.

## Data Availability

Data are provided in the electronic supplementary material [40], and through the LACM-DI collections database.
